# Changes in mindful eating and eating behaviors among female university students taking nutrition courses

**DOI:** 10.1186/s40337-026-01624-8

**Published:** 2026-05-04

**Authors:** Sine Yilmaz, Pınar Göbel, Zeynep Bengisu Ejder, Şule Kocabaş, Büşra Açikalin Göktürk, Başak Can, Selen Müftüoğlu, Nevin Şanlier

**Affiliations:** 1 Faculty of Health Sciences, Department of Nutrition and Dietetics, Ankara Medipol University, Ankara, Turkey; 2 Gülhane Faculty of Health Sciences, Department of Nutrition and Dietetics, Health Science University, Ankara, Turkey; 3 Faculty of Health Sciences, Department of Nutrition and Dietetics, Istanbul Beykent University, Istanbul, Turkey

**Keywords:** Nutrition education, Mindful eating, Emotional eating, Restrictive eating, External eating

## Abstract

**Background:**

This research aimed to investigate the impact of a structured nutrition education program on the dietary behaviours and mindful eating practices of university students. Given that the university phase is critical for developing healthy lifestyle choices, such initiatives show an important opportunity for public health improvement.

**Methods:**

A comparative quasi-experimental design was utilized, featuring both experimental and control groups exclusively comprising female students. The experimental group participated in a 14-week structured nutrition education program that integrated interdisciplinary content. The curriculum covered fundamental nutrition concepts, nutritional needs throughout different life stages, physical activity, food science, and principles of healthy eating behaviors. Each session reinforced behavioral awareness through discussions of relevant scientific literature. Pre- and post-intervention assessments were conducted.

**Results:**

At the conclusion of the program, the total score on the Dutch Eating Behavior Questionnaire (DEBQ) within the experimental group declined from 2.85 to 2.44; specifically, scores for external eating fell from 2.81 to 2.29, and emotional eating scores decreased from 3.18 to 2.59—each showing statistically significant reductions (*p* < 0.001). Conversely, the Mindful Eating Questionnaire (MEQ) score improved from 2.75±0.40 to 3.80±0.39 (*p* < 0.001). In contrast, no notable changes were recorded in either DEBQ or MEQ scores among participants in the control group (*p* > 0.05).

**Conclusions:**

A structured 14-week nutrition education program was associated with improved mindful eating and lower emotional, external, and restrained eating scores among female university students These findings underscore the positive transformations stemming from increased cognitive awareness, particularly regarding external and emotional eating tendencies. Nutrition interventions at universities appear to be an effective strategy for promoting both individual well-being and community health. Furthermore, these results highlight that effective nutrition education empowers individuals to integrate their knowledge into everyday practices. Therefore, it is essential to implement evidence-based methods and resources for nutrition education across all age groups and backgrounds.

**Graphical Abstract:**

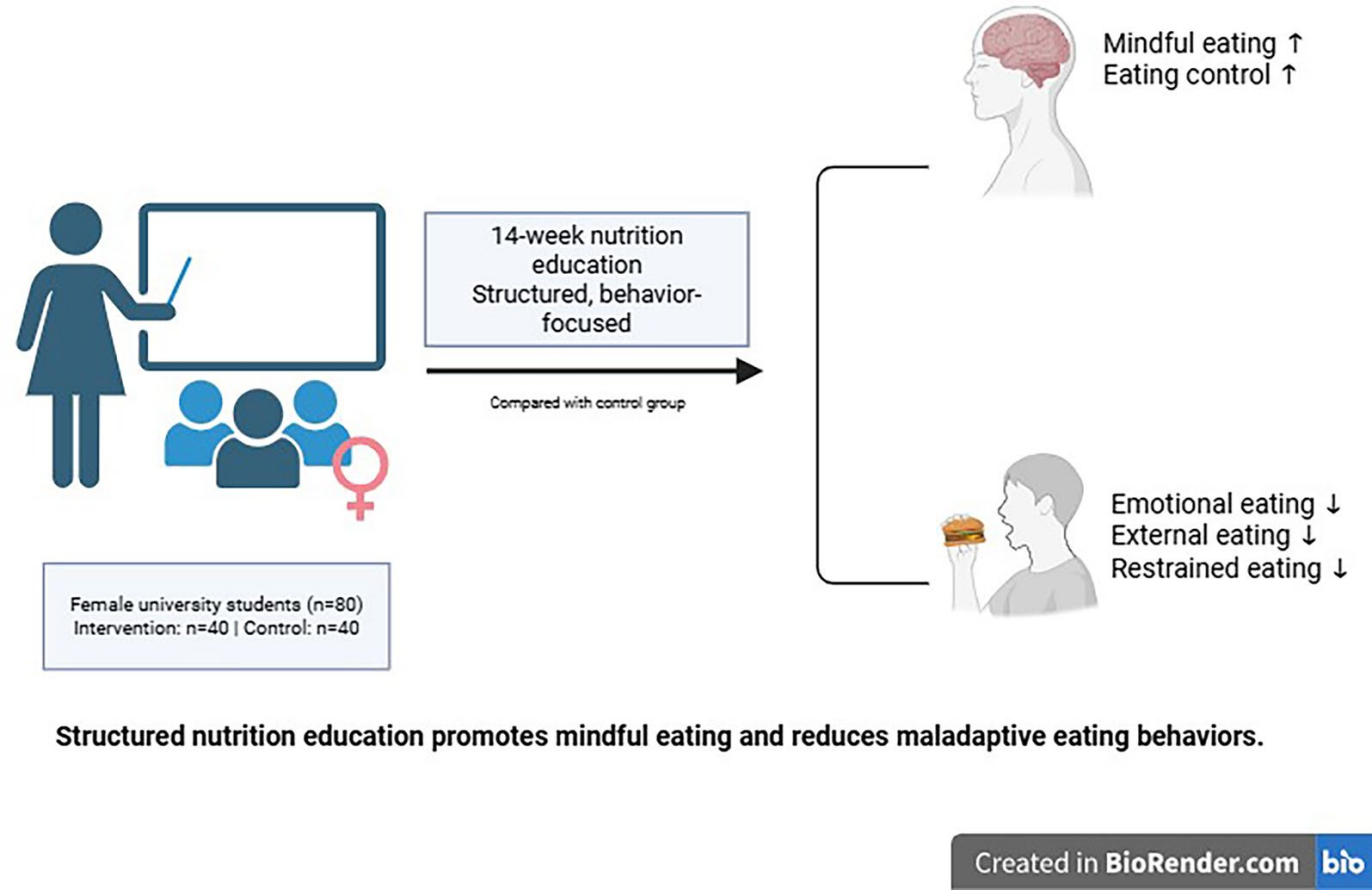

**Supplementary Information:**

The online version contains supplementary material available at 10.1186/s40337-026-01624-8.

## Abstract (plain language summary)

University years are a time when eating habits can change because of stress, new routines, and changes in the social environment. This study examined whether a 14-week elective nutrition course could help female university students become more aware of their eating behaviors. A total of 71 students took part in the study: 38 students attended the nutrition course and 33 students did not. Students completed questionnaires before and after the semester. After the course, students who attended the nutrition education program reported higher mindful eating scores and lower scores for emotional, external, and restrained eating. In other words, they appeared to become more aware of hunger and fullness cues and reported fewer eating behaviors driven by emotions, environmental food cues, or restriction. These changes were not observed in the comparison group.The findings suggest that a structured nutrition education course may support healthier and more regulated eating behaviors among female university students. However, this study does not show that nutrition education prevents eating disorders. The results should also be interpreted carefully because the study included only female students from one university, used self-report questionnaires, and did not include long-term follow-up. Future studies with larger and more diverse groups are needed to see whether these changes continue over time.

**Supplementary Information:**

The online version contains supplementary material available at 10.1186/s40337-026-01624-8.

## Introduction

Eating habits that support physical and mental wellbeing are important for maintaining health and preventing disease. Nutrition is critical for disease prevention, maintaining health, and improving quality of life. In this context, structured nutrition education is an effective way to inform individuals about healthy eating and raise awareness. Nutrition education encompasses systematic and goal-oriented learning processes that support individuals in voluntarily adopting healthy eating habits [1, 2].

Mindful eating refers to an individual’s ability to regulate food intake independently of emotional and environmental triggers, considering physical cues such as hunger, fullness, and taste [3, 4]. Increased mindful eating enables individuals to question unhealthy eating behaviors and make more conscious choices. Educating individuals to develop this awareness is considered an important step in improving eating behavior [5].

Improving eating behavior is important, as maladaptive eating patterns such as emotional, external, and restrained eating have been associated with dysregulated energy intake, weight-related concerns, and poorer psychological wellbeing. These patterns may also increase vulnerability to disordered eating tendencies, particularly during transitional life periods such as young adulthood [6].

The transition to university is a period of rapid change in lifestyle and dietary habits. Research shows that the vast majority of eating disorders begin between the ages of 18 and 25, and a significant proportion of these individuals are university students (e.g., disordered eating rates are approximately 20% in global meta-analyses and 38–48% in some post-pandemic studies) [7, 8]. Therefore, the university period is strategically considered for healthy eating education and awareness intervention. The prevalence of emotional eating behavior among university students ranges from 20% to 50%, with higher rates reported among female students. The literature consistently shows that body mass index, body dissatisfaction, and perceived stress levels are significantly associated with emotional eating [9–11]. Changes in lifestyle associated with transitioning to university, such as a new social environment, living away from family, increased academic stress, and increased consumption of meals outside the home, can negatively affect eating behaviors [12, 13]. Previous studies have reported that individuals who received nutrition education showed an increase in their knowledge levels, but this increase did not always translate into behavioral change [14, 15]. Rababah et al. [16] observed a decrease in emotional eating and uncontrolled eating scores and an increase in mindful eating after a 3-month motivational interviewing-based intervention for female university students. While previous studies have primarily focused on improving nutrition knowledge, evidence suggests that knowledge alone may not be sufficient to change eating behaviors. Therefore, the present study aimed to evaluate the impact of a structured, behavior-focused 14-week nutrition education course on both mindful eating and eating behavior patterns. Unlike prior studies, this intervention was embedded within a university curriculum and emphasized awareness-based and behavioral components alongside knowledge, providing a more comprehensive approach to eating behavior change.

It is important to note that public messaging around eating may, in some cases, unintentionally contribute to rigid or dichotomous thinking about food, which has been linked to disordered eating tendencies. Therefore, nutrition education approaches should aim to promote flexible, behavior-focused, and awareness-based eating patterns, rather than prescriptive or restrictive rules [17].

Female university students are more sensitive than male students in terms of eating behaviors and body image due to social norms, the influence of social media, and cultural expectations regarding body image [9, 11]. Behaviors such as weight control, dieting, and emotional eating are more prevalent among women, making them more receptive and responsive to interventions such as nutrition education [10]. Furthermore, female students have higher levels of body dissatisfaction, social comparison, and susceptibility to eating disorders, allowing them to benefit more from education on mindful eating [1]. Therefore, the study’s focus on female university students can be considered a meaningful and targeted choice in a social context.

Nutrition education extends beyond short-term outcomes and may support the development of a more sustainable and balanced relationship with food over time. In this context, the present study evaluated the effects of a one-semester structured, behavior-focused nutrition education program by comparing female university students who received the education with those who did not. The aim of this study was to evaluate the effect of a structured nutrition education program on mindful eating and eating behavior patterns in female university students.

## Method

**Fig. 1:**
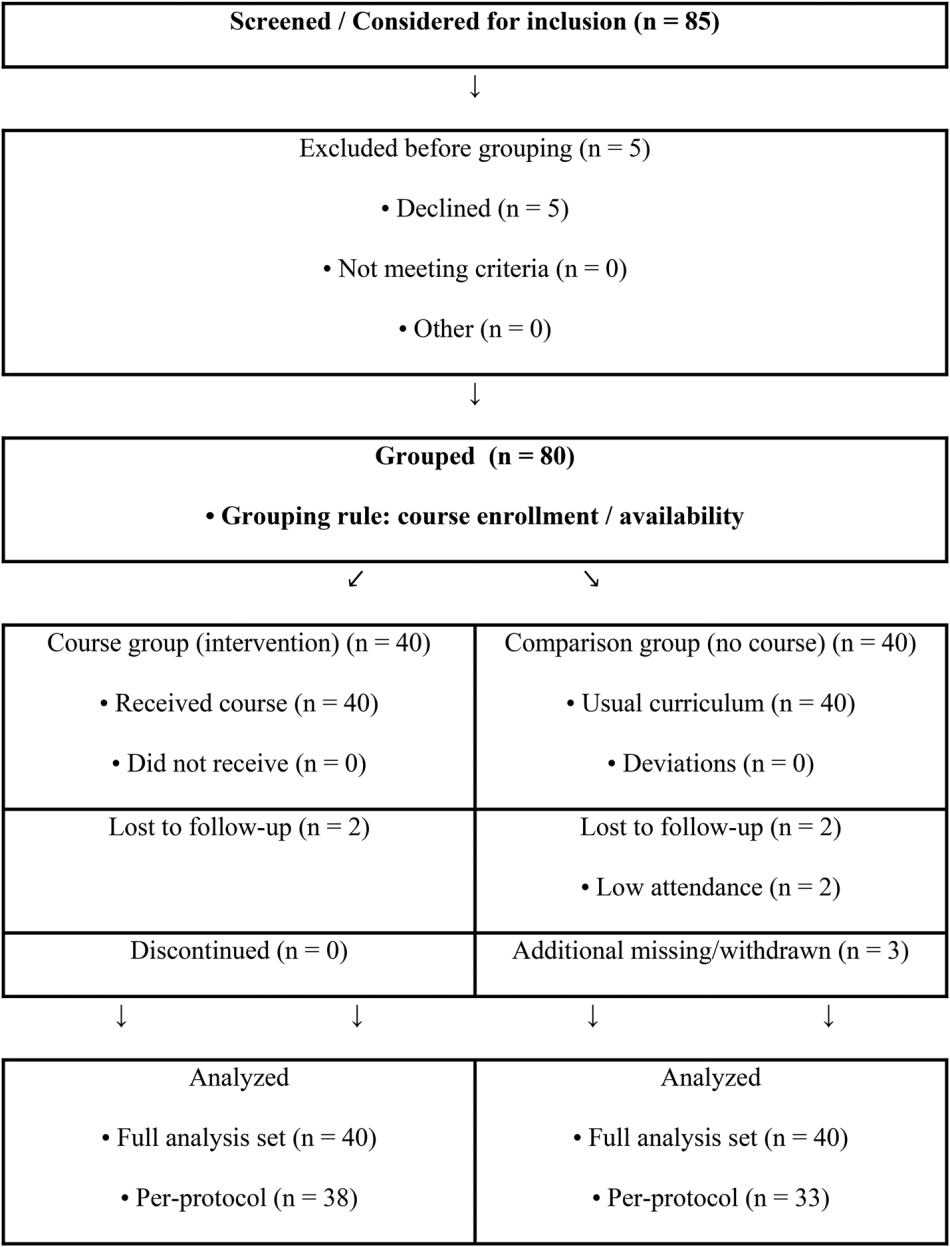
Participant flow diagram throughout the study (TREND)

This was a quasi-experimental controlled study conducted at Ankara Medipol University in Türkiye, comparing students who took a 14-week *Introduction to Human Nutrition (elective)* course with those who did not. This study was reported in accordance with the TREND (Transparent Reporting of Evaluations with Nonrandomized Designs) guidelines, which provide a framework for transparent reporting of non-randomized intervention studies [18]. Participants were grouped according to course enrollment/availability into an Intervention group (*n* = 40) and a Comparison group (*n* = 40). This study was conducted using a quasi-experimental design with an intervention and a comparison group, with measurements taken at baseline and post-intervention in both groups. The intervention group consisted of female university students who voluntarily enrolled in a 14-week elective nutrition course. The comparison group included female students from the same department who did not enroll in the course during the same academic period. Participants were not randomly assigned to groups, as course enrollment was based on students’ own preferences. However, the groups had similar academic backgrounds and comparable baseline characteristics, including academic performance. Eligibility criteria were: never having taken any nutrition courses; no psychiatric history; voluntary consent; and active enrollment at Ankara Medipol University where attendance is compulsory. Psychiatric history, including eating disorders, was assessed based on participants’ self-report through the general information form, and no clinical diagnostic evaluation was conducted. Students from the Department of Nutrition and Dietetics (i.e., those with prior formal nutrition education), those who missed more than four weeks of classes, and those who self-reported a current eating disorder were considered ineligible for inclusion. However, no participant reported a current eating disorder. To minimize gender imbalance (given the very small number of males in the course cohort), only female students were recruited. Figure 1 presents the participant flow diagram. Primary analyses were conducted using a per-protocol (complete-case) approach including participants who provided both baseline and post-intervention data (Intervention *n* = 38; Comparison *n* = 33). Missing data were not imputed.

### Course content: introduction to human nutrition (elective)

The experimental group studied the course “Introduction to Human Nutrition (elective)”, during 14 weeks (two hours per week). It aimed at sharing basic nutrition knowledge in terms of nutrition’s principles, clarifying the role and health effect of macronutrients and micronutrients, and demonstrating nutritional needs during different stages of life from pregnancy, childhood to adolescence, adulthood and old age. It also considered the trajectory of eating behaviours and associated health problems. Following each session, students participated to critically assess scientific papers related to recent eating behavior literature in order to improve scientific literacy and critical thinking skills.

The course included both theoretical nutrition content and behavior-focused components aimed at improving eating awareness and self-regulation. Topics were delivered using an interactive approach and included discussions on hunger and satiety cues, emotional and external eating, and mindful eating practices. A detailed outline of the weekly course content is provided in Supplementary Table S1.

### Data collection process

The data were collected using face-to-face surveys. Survey forms were provided to consenting students during the first class of the semester and administered to them in class. A unique code was chosen by the participants to secure their anonymity. The same sets of questionnaires were re-administered at week 14 (end-of-semester) to collect post-test data. These codes were used to match baseline and post-intervention responses. Written informed consent for the study was obtained from all participants prior to data collection.

This study had two stages, first to compare pre-test data between groups (students in nutrition course and students not taking the class) as a baseline characteristic for subjects; and secondly to compare changes within the group of students who participated in the nutrition course at the final stage of the semester (the intervention effect). This study was approved by the Ankara Medipol University Non-Interventional Ethics Committee (Ethics Committee No: 188) and conducted in accordance with ethical standards specified in the World Medical Association’s Declaration of Helsinki.

### Data collection

Four main sections comprised of the tools used in collecting of data.

General Information Form: This form contained questions on general characteristics and lifestyle, including age, grade year in school, diet practice, physical activity level and parental education. Dietary practices were assessed through self-reported questions regarding meal patterns, dieting behaviors, and any current attempts to modify eating habits. Participants were classified as physically active if they reported engaging in at least 150 min of moderate-intensity physical activity per week, in accordance with World Health Organization recommendations; otherwise, they were classified as inactive [19].

Anthropometric Measurements: Body mass (kg) measurement was done in controlled conditions on an empty stomach for the morning session, with participants dressed lightly and without shoes using a calibrated scale accurate up to 0.5 kg. Height (cm) was measured using a portable stadiometer accurate to 0.1 cm with participants standing with their feet close together and head in the correct position according to Frankfort plane guidelines [20]. Body Mass Index (BMI) was calculated as the ratio of weight (kg)/height²(m²). According to WHO recommendations based on 2004 criteria, BMIs were classified as underweight (< 18.5 kg/m²), normal/healthy weight (18.5–24.9 kg/m²) and overweight/obese (≥ 25 kg/m²) [21].

Dutch Eating Behavior Questionnaire (DEBQ): It was developed by Van Strien et al., and consists of 33 items under three subdimensions; restrained eating (Items 1–10), emotional eating (Item 11–23) and external eating (Item 24-33); then translated into Turkish by Bozan in 2009. Responses are rated on a five-point Likert scale, running from “never” (1) to “very often” (5), and also contain an alternative “not applicable” [22, 23]. Item 31 in external eating is reverse-scored; total scores are not summed but run per subscale separately, higher scores reflect poorer eating behaviour.

Mindful Eating Questionnaire (MEQ): This scale was developed by Framson *et* al. [24] and adapted into Turkish by Köse *et* al. [25]. The scale consists of 30 items and seven subscales: Disinhibition, Emotional Eating, Eating Control, Focus, Eating Discipline, Mindfulness, and Interference. Disinhibition reflects loss of control over eating and overeating tendencies; Emotional Eating refers to eating in response to emotional states; Eating Control assesses the ability to regulate eating behavior; Focus represents attention to the sensory experience of eating; Eating Discipline reflects structured and planned eating habits; Mindfulness refers to awareness of internal cues such as hunger and satiety; and Interference captures the influence of external or environmental factors that may disrupt eating awareness. It uses a 5-point Likert-type rating system. High scores indicate higher mindful eating in that subdimension. In addition to the subdimensions, a mindful eating score was obtained. When scoring the scale, the average of the sub-dimensions and the total score is considered [25]. As the score obtained from the scale increases, mindful eating increases. All factor scores obtained from the scale were evaluated positively. For example, individuals who score high on the emotional eating dimension are considered to have high coping skills for emotional eating. In general, it can be said that as the score obtained from the scale increases, individuals’ levels of mindful eating also increase.

### Statistical analysis

The SPSS software (version 26) was used to manage data sets, calculate descriptive statistics, and analyze baseline differences between groups. Independent samples t-tests were applied for continuous variables (age, height, body weight, and BMI), Fisher’s exact test for binary categorical variables (working status, diet status, and physical activity), and the Monte Carlo exact test for categorical variables with more than two categories (mother’s and father’s education). Pre-test scores between experimental and control groups were compared using independent samples t-tests (for normally distributed data, as also for post-test). To analyze between group differences, paired samples t-tests were used to compare pre- and post-test results among groups. In addition, eta squared values were used to interpret the practical significance according to Cohen’s cut-off points: small effect η² ≥0. 01, medium effect η² ≥0. 06, large effect η² ≥0. 14. Type I error threshold was *p* < 0. 05 throughout all analyses conducted [26].

## Results

A total of 71 female students participated in the study, including 38 assigned to the experimental group (mean age: 20.45 ± 1.88 years) and 33 in the control group (mean age:19.06 ± 0.78 years). All participants were between the ages of 18 and 22. Baseline characteristics of the participants are presented in Table 1. Significant between-group differences were observed for age (*p* < 0.001) and height (*p* = 0.047), whereas no significant differences were found for body weight or BMI (*p* > 0.05).

**Table 1:** Participant characteristics and between-group comparisons at baseline

Demographic information	Experimental group (*n* = 38)	Control group (*n* = 33)	*p* value
	*n* (%)	*n* (%)	
Working Status			
Working	5 (13.2)	0 (0)	0.056^a^
Not working	33 (86.8)	33 (100.0)	
Dietary Status			
Dietary restriction	1 (2.6)	4 (12.1)	0.176^***a***^
No restriction	37 (97.4)	29 (87.9)	
Physical Activity			
Active	21 (55.3)	18 (54.5)	1.00^**a**^
Inactive	17 (44.7)	15 (45.5)	
Mother’s highest level of education			
Primary School	8 (21.1)	19 (57.6)	0.09^***b***^
High School	19 (50.0)	11 (33.3)	
University	11 (28.9)	3 (9.1)	
Father’s highest level of education			
Primary School	3 (7.9)	16 (48.4)	***< 0.001*** ^***b***^
High School	17 (44.7)	10 (30.3)	
University	18 (47.4)	7 (21.2)	
Age (years)	20.45 ± 1.88 (19– 22)	19.06 ± 0.78 (18–21)	***< 0.001*** ^*c*^
Height (cm)	167.32 ± 5.3 (158.0–180.0)	164.66 ± 5.67 (155.0–174)	0.047^***c***^
Body weight (kg)	62.42 ± 12.02 (49.0–90.0)	59.21 ± 10.92 (42.0–91.0)	0.212^***c***^
BMI (kg/m²)	22.32 ± 3.69 (17.1–30.0)	21.84 ± 3.97 (17.48–33.20)	0.606^***c***^

*p* < 0.001, ^a^Fisher’s exact test; ^b^Monte Carlo exact test; ^c^independent samples t-test were used in statistical analyses

Table 2 presents the pre- and post-test DEBQ and MEQ scores for the intervention and comparison groups, including within-group and between-group comparisons.There were no between-group differences in the entire DEBQ and MEQ scores at baseline (*p* > 0.05). Following the intervention, the intervention group showed significantly lower total and subscale DEBQ scores and significantly higher total and subscale MEQ scores compared with the comparison group (*p* < 0.001). Within-group analyses showed that DEBQ scores decreased and MEQ scores increased significantly over time in the intervention group, whereas no significant pre-post differences were found in the comparison group, except for the Interference subscale. Effect sizes for between-group comparisons ranged from moderate to large, with large effects observed for total DEBQ (η² = 0.30) and total MEQ (η² = 0.43) scores.

**Table 2:** Within- and between-group comparisons of DEBQ and MEQ subscale scores (mean ± SD)

Scale	Subscale	Exp. Pre	Exp. Post	Ctrl. Pre	Ctrl. Post	*p* ^a^ (within Exp.)	η²^a^ (within Exp.)	*p* ^b^ (within Ctrl.)	η²^b^ (within Ctrl.)	*p*^c^ (between pre)	η²^c^ (between pre)	*p*^d^ (between post)	η²^d^ (between post)
**DEBQ**	Emotional Eating	2.56 ± 1.10	1.61 ± 0.55	2.38 ± 0.86	2.37 ± 0.88	***< 0.001***	0.37	0.98	–	0.44	–	***< 0.001***	0.22
	Restrained Eating	2.83 ± 0.92	2.02 ± 0.64	2.81 ± 1.04	2.52 ± 0.97	***< 0.001***	0.34	0.06	–	0.94	–	***< 0.001***	0.09
	External Eating	3.19 ± 0.82	2.46 ± 0.64	3.24 ± 0.78	3.22 ± 0.89	***< 0.001***	0.34	0.93	–	0.81	–	***< 0.001***	0.20
	Total	2.86 ± 0.65	2.02 ± 0.39	2.81 ± 0.53	2.70 ± 0.64	***< 0.001***	0.50	0.35	–	0.72	–	***< 0.001***	0.30
**MEQ-30**	Disinhibition	2.59 ± 0.88	3.70 ± 0.82	2.70 ± 0.65	2.74 ± 0.71	***< 0.001***	0.55	0.75	–	0.56	–	***< 0.001***	0.28
	Emotional Eating	2.79 ± 1.14	3.86 ± 0.88	2.93 ± 0.83	2.90 ± 0.85	***< 0.001***	0.41	0.80	–	0.55	–	***< 0.001***	0.27
	Eating Control	2.36 ± 0.60	3.43 ± 0.57	2.52 ± 0.72	2.54 ± 0.81	***< 0.001***	0.71	0.86	–	0.33	–	***< 0.001***	0.30
	Focus	3.65 ± 0.59	4.42 ± 0.43	3.82 ± 0.65	3.90 ± 0.66	***< 0.001***	0.51	0.50	–	0.25	–	***< 0.001***	0.19
	Eating Discipline	3.14 ± 0.59	4.19 ± 0.57	3.29 ± 0.76	3.31 ± 0.79	***< 0.001***	0.60	0.85	–	0.38	–	***< 0.001***	0.30
	Mindfulness	2.51 ± 0.52	3.61 ± 0.58	2.53 ± 0.61	2.48 ± 0.56	***< 0.001***	0.63	0.53	–	0.90	–	***< 0.001***	0.50
	Interference	2.24 ± 0.88	3.42 ± 0.93	2.30 ± 0.96	2.71 ± 0.86	***< 0.001***	0.47	***< 0.001***	0.25	0.76	–	***< 0.001***	0.14
	Total	2.75 ± 0.40	3.80 ± 0.39	2.87 ± 0.35	2.94 ± 0.45	***< 0.001***	0.73	0.22	–	0.20	–	***< 0.001***	0.43

Effect sizes (η²) were calculated for both within-group and between-group comparisons. Values of 0.01, 0.06, and 0.14 indicate small, medium, and large effects, respectively. *p*^*a*^ : *within experimental group; p*^*b*^: *within control group; p*^*c*^: *between pre experimental and control group; p*^*d*^: *between post experimental and control*

## Discussion

This study aimed to evaluate the effect of a structured nutrition education program on mindful eating and eating behavior patterns in female university students. The findings demonstrated significant differences in self-reported mindful eating and eating behavior scores between the experimental and control groups following the intervention. These results suggest that participation in a structured, behavior-focused nutrition education program may be associated with improvements in eating-related outcomes among young adults. Importantly, the present study does not aim to directly address eating disorder prevention; rather, it focuses on promoting awareness and regulation of eating behaviors within a general population.

Nutrition education is considered a critical intervention area for individuals adopting healthy lifestyles, preventing diseases associated with inadequate and unbalanced nutrition, and improving health [27]. The university period is a sensitive time when individuals’ lifelong nutrition habits are shaped, and psychosocial factors such as academic pressure, changes in social environment, and individual independence have a decisive impact on eating behaviors [28]. This study found significant increases in mindful eating levels and positive changes in eating attitudes among female university students following a 14-week “Introduction to Human Nutrition (elective)” course.

Although the present findings are consistent with previous intervention studies reporting improvements in mindful eating and eating behaviors [29–31], this study contributes to the literature in several important ways. Unlike many prior studies that primarily focused on short-term interventions or knowledge-based approaches [31, 33], the present study evaluated a structured 14-week course embedded within a university curriculum and emphasized behavior-focused and awareness-based components. Furthermore, the simultaneous assessment of both mindful eating (MEQ) and eating behavior patterns (DEBQ) provides a more comprehensive understanding of eating-related outcomes. In addition, the study offers context-specific evidence from a Turkish university population, which remains underrepresented in the existing literature.

Nutrition education has the potential to convey theoretical knowledge and create lasting changes in individuals’ attitudes, self-efficacy, and behaviors [32]. In the current study, increased mindful eating scores and decreased total DEBQ scores suggest favorable changes in self-reported eating-related outcomes following the intervention. Notably, the education was implemented during a highly stressful period, such as the final exam week, demonstrating that it can be effective even under stress. Similar findings have been reported in the literature. Park *et* al. [33] observed positive effects on portion control, self-efficacy, and emotional eating in university students following a 12-week nutrition awareness training program. A meta-analysis by Yue *et* al. [31] indicated that nutrition education programs for university students produced significant effects in many dimensions, such as mindful eating, healthy food selection, and portion control. Psychosocial education-based interventions have likewise been reported to reduce emotional and external eating tendencies and improve mindful eating [30, 34].

The university period may be particularly relevant for nutrition education because eating behaviors are shaped by both environmental and psychosocial influences during this stage of life. Previous studies suggest that nutrition education during this period may support not only short-term improvements but also the development of longer-term eating-related behaviors [27, 35]. However, studies directly measuring changes in eating behavior are relatively limited [36]. Boek *et* al. [37] found that the key factors influencing nutrition behaviors in female university students are social influence, self-esteem, and stress levels, which can be positively influenced by a well-structured nutrition education program. Similarly, Tazeoğlu *et* al. [30] reported a decrease in emotional and external eating scores and a significant increase in mindful eating scores in an eight-week psychosocial education-based program. While similar improvements in eating-related outcomes have been reported in previous studies [30], the findings of the present study extend this evidence by demonstrating that such changes can be observed within a structured, curriculum-based intervention over a longer duration. Therefore, rather than presenting entirely novel outcomes, the study strengthens existing evidence by providing a comprehensive and context-specific evaluation of behavioral changes in a real-life educational setting.

Baseline DEBQ and MEQ scores did not differ significantly between the intervention and comparison groups, suggesting that the groups were broadly comparable in terms of eating-related attitudes and behaviors at study entry. After the intervention, significant improvements were observed only in the intervention group. In particular, total DEBQ scores decreased from 2.86 ± 0.65 to 2.02 ± 0.39, whereas total MEQ scores increased from 2.75 ± 0.40 to 3.80 ± 0.39. These changes were observed despite the post-test assessment being conducted close to the final exam period, when stress levels may have been elevated. An increase in mindful eating indicates that individuals are better able to recognize hunger and satiety cues, cope with emotional eating, and make more conscious food choices. These findings are consistent with previous research showing that mindfulness-and mindful eating-based intervention programs can reduce emotional and external eating behaviors [29, 34, 38]. In contrast, the fact that the positive changes achieved were maintained even during a stressful period suggests that this training may have been internalized not only through cognitive processes but also through behavioral learning and neuropsychological regulation processes [39]. Overall, the results suggest that structured nutrition education may help strengthen internal eating regulation in young adults.

The intervention in this study extended beyond basic nutrition knowledge by incorporating multidisciplinary content, including life-cycle nutrition, physical activity, food science, and food selection. In addition, the discussion of a scientific article at the end of each lesson may have supported students’ scientific literacy, critical thinking, and ability to relate theoretical knowledge to behavioral awareness. This broader educational approach may have contributed to the observed changes by facilitating both cognitive understanding and reflective engagement with eating-related issues. Previous studies have similarly shown that structured nutrition education programs can improve diet quality, fruit and vegetable intake, physical activity, and nutrition literacy among university students [40, 41].

Nutrition education is not limited to the transfer of cognitive knowledge; it also enables individuals to recognize their eating behaviors, transform unhealthy patterns, and develop awareness of healthy eating. The literature indicates that mindful eating-based interventions facilitate individuals’ ability to better recognize hunger and fullness cues, develop resilience against emotional triggers, and make healthy choices [3, 42]. For example, Rababah *et* al. [16] reported that nutrition education supported by motivational interviewing reduced emotional and uncontrolled eating levels while increasing mindful eating behaviors among female university students. Similarly, Warren *et* al. [43] [emphasized that mindful eating training is effective in reducing external eating behaviors. Furthermore, it is suggested that such interventions should be supported by reinforcing components, such as regular follow-up, application examples, and feedback, to ensure that their effects are not limited to the short term and that they can create sustainable behavioral change in the long term [44, 45]. However, in the current study, despite the final test being administered during a period of high stress for students, such as the final exam week, external eating scores in the experimental group decreased significantly from 3.19 ± 0.82 to 2.46 ± 0.64, and emotional eating scores decreased significantly from 2.56 ± 1.10 to 1.61 ± 0.55. In contrast, the literature indicates that high stress levels increase emotional and external eating behaviors [10, 46, 47]. These findings suggest that structured nutrition education may support the regulation of eating behaviors even under challenging conditions.

The Society for Nutrition Education and Behavior (SNEB) states that nutrition education aimed at improving the health of individuals, communities, and food systems should meet six core competencies: basic food and nutrition knowledge, nutrition throughout the life cycle, food science, physical activity, food and nutrition policies, and agricultural production and food systems [48]. The education program implemented in the current study was planned to cover a large part of these competencies and succeeded in increasing the participants’ theoretical knowledge, cognitive awareness, and behavioral transformation. These findings support the view that structured nutrition education may represent a promising strategy for promoting eating-related awareness and healthier behavior patterns among university students.

University years represent a period of transition in which eating behaviors are shaped by both environmental and psychosocial factors. Nutrition education during this time may support the development of sustainable eating habits by enhancing individuals’ ability to manage emotional, social, and behavioral influences on food choices [49]. Nutrition education during this period may support the development of more sustainable eating habits by helping individuals better manage emotional, social, and behavioral influences on food choices. Since eating-related patterns established in early adulthood may persist over time and carry important individual and public health implications [50], the university setting may provide a valuable opportunity for behavior-focused nutrition education. In this context, the present findings suggest that a structured nutrition education program may support improvements in mindful eating and eating-related outcomes among female university students, even when assessed during a potentially stressful academic period.

## Limitations

This study has several limitations. First, it was conducted at a single university and included only female students; therefore, the generalizability of the findings is limited. Future studies involving larger and more diverse samples are needed to assess whether similar results can be observed in other student populations. Second, the study relied solely on self-report measures, which may not fully reflect actual eating behaviors and may be subject to reporting bias. Third, the sustainability of the observed changes could not be evaluated, as no long-term follow-up was conducted. In addition, the potential influence of the instructor’s communication style, motivation, and feedback was not systematically assessed, although these factors may have contributed to the educational process. Finally, because the study used a quasi-experimental design without random assignment, causal inferences should be made with caution. Nevertheless, the findings provide useful insight into eating-related changes observed within a real-life educational setting.

## Conclusion and future directions

This study suggests that structured nutrition education may be associated with favorable changes in eating-related outcomes and mindful eating among female university students. The findings indicate that the educational process may have supported not only knowledge acquisition but also greater awareness of eating-related behaviors. In particular, the decreases observed in emotional and external eating scores together with the increase in mindful eating scores suggest that nutrition education may contribute to more adaptive eating-related patterns.

Given that nutrition education is a dynamic process, future interventions may benefit from continuity, reinforcement, and follow-up in order to support the maintenance of these changes over time. More broadly, nutrition education may represent a valuable tool for promoting healthier lifelong habits, particularly when delivered during transitional life stages such as university. Incorporating interdisciplinary and behavior-focused programs into university curricula, and grounding educational content in established behavior change theories such as Cognitive Behavioral Therapy or the Transtheoretical Model may further strengthen the impact of such interventions.

## Electronic Supplementary Material

Supplementary Material 1.

Supplementary Material 2.

## Data Availability

All data analyzed are presented in this article.

## Abbreviations

DEBQ: Dutch Eating Behavior Questionnaire
MEQ: Mindful Eating Questionnaire
BMI: Body Mass Index

## References

[1] Murimi MW, Kanyi M, Mupfudze T, Amin MR, Mbogori T, Aldubayan K. Factors influencing efficacy of nutrition education interventions: a systematic review. J Nutr Educ Behav. 2017;49(2):142–65. 10.1016/j.jneb.2016.09.003. 27814976 10.1016/j.jneb.2016.09.003

[2] Swindle T, Curran GM, Johnson SL. Implementation science and nutrition education and behavior: opportunities for integration. J Nutr Educ Behav. 2019;51(6):763–74. 10.1016/j.jneb.2019.02.009. 30982567 10.1016/j.jneb.2019.03.001 PMC6904925

[3] Van Dyke N, Drinkwater EJ. Relationships between intuitive eating and health indicators: a literature review. Public Health Nutr. 2014;17(8):1757–66. 10.1017/S1368980013002139. 23962472 10.1017/S1368980013002139 PMC10282369

[4] Lyzwinski LN, Edirippulige S, Caffery L, Bambling M. Mindful eating mobile health apps: review and appraisal. JMIR Ment Health. 2019;6(8):e12820. 10.2196/12820. 31441431 10.2196/12820 PMC6727629

[5] Paolassini-Guesnier P, Van Beekum M, Kesse-Guyot E, et al. Mindful eating is associated with a healthier plant-based diet in the NutriNet-Santé study. Sci Rep. 2025;15:19928. 10.1038/s41598-025-02195-5. 40481021 10.1038/s41598-025-02195-5 PMC12144130

[6] Hootman KC, Guertin KA, Cassano PA. Stress and psychological constructs related to eating behavior are associated with anthropometry and body composition in young adults. Appetite. 2018;125:287–94. 10.1016/j.appet.2018.01.003. 29309851 10.1016/j.appet.2018.01.003 PMC5878735

[7] Alhaj OA, Fekih-Romdhane F, Sweidan DH, et al. The prevalence and risk factors of screen-based disordered eating among university students: a global systematic review, meta-analysis, and meta-regression. Eat Weight Disord. 2022;27:3215–43. 10.1007/s40519-022-01452-0. 35925546 10.1007/s40519-022-01452-0 PMC9362208

[8] Pacanowski CR, Skubisz C, Borton D, et al. Prevalence and correlates of disordered eating at a large state university before and after the onset of the COVID-19 pandemic. J Eat Disord. 2024;12:153. 10.1186/s40337-024-01056-2. 39354601 10.1186/s40337-024-01056-2 PMC11446083

[9] Shatwan IM, Alzharani MA. Association between perceived stress, emotional eating, and adherence to healthy eating patterns among Saudi college students: a cross-sectional study. J Health Popul Nutr. 2024;43:144. 10.1186/s41043-024-00637-w. 39252087 10.1186/s41043-024-00637-w PMC11385838

[10] Zhou J, Chen Y, Ji S, et al. Sleep quality and emotional eating in college students: a moderated mediation model of depression and physical activity levels. J Eat Disord. 2024;12:155. 10.1186/s40337-024-01107-8. 39375757 10.1186/s40337-024-01107-8 PMC11460174

[11] Alexatou O, Papadopoulou SK, Mentzelou M, Deligiannidou GE, Dakanalis A, Giaginis C. Exploring the impact of emotional eating among university students: a literature review. Med Sci. 2025;13(2):56. 10.3390/medsci13020056. 10.3390/medsci13020056 PMC12101398 40407551

[12] Maillet MA, Grouzet FME. Understanding changes in eating behavior during the transition to university from a self-determination theory perspective: a systematic review. J Am Coll Health. 2023;71(2):422–39. 10.1080/07448481.2021.1891922. 34292133 10.1080/07448481.2021.1891922

[13] Almoraie NM, Alothmani NM, Alomari WD, Al-Amoudi AH. Addressing nutritional issues and eating behaviours among university students: a narrative review. Nutr Res Rev. 2025;38(1):53–68. 10.1017/S0954422424000088. 38356364 10.1017/S0954422424000088

[14] Kızıltan G. Başkent Üniversitesi yiyecek içecek işletmeciliği programına kayıtlı öğrencilerin beslenme bilgi düzeyi ve beslenme durumuna beslenme eğitiminin etkisi. Beslenme ve Diyet Derg. 2000;29(2):34–41.

[15] Devran BS, Saka M. Lise öğrencilerine verilen beslenme eğitiminin beslenme alışkanlıkları, beslenme bilgi düzeyi ve fiziksel aktivite üzerine etkisi. Beslenme ve Diyet Derg. 2019;47(3):5–14.

[16] Rababah J, Al-Hammouri MM. Effect of a modified motivational interviewing intervention on university students’ psychological, cognitive, and nutritional health: a randomized controlled trial. Nurs Forum. 2022;57(6):1424–33. 10.1111/nuf.12773. 36380519 10.1111/nuf.12841

[17] Linardon J, Mitchell S. Rigid dietary control, flexible dietary control, and intuitive eating: evidence for their differential relationship to disordered eating and body image concerns. Eat Behav. 2017;26:16–22. 10.1016/j.eatbeh.2017.01.008. 28131005 10.1016/j.eatbeh.2017.01.008

[18] Des Jarlais DC, Lyles C, Crepaz N, TREND Group. Improving the reporting quality of nonrandomized evaluations of behavioral and public health interventions: the TREND statement. Am J Public Health. 2004;94(3):361–6. 10.2105/ajph.94.3.361. 14998794 10.2105/ajph.94.3.361 PMC1448256

[19] World Health Organization. Global recommendations on physical activity for health. Geneva: WHO; 2010. 26180873

[20] Lohman TG, Roche AF, Martorell R. Anthropometric standardization reference manual. Champaign: Human Kinetics Books; 1988.

[21] WHO Expert Consultation. Appropriate body-mass index for Asian populations and its implications for policy and intervention strategies. Lancet. 2004;363:157–63. 10.1016/S0140-6736(03)15268-3. 14726171 10.1016/S0140-6736(03)15268-3

[22] Van Strien T, Frijters JE, Bergers GP, Defares PB. The Dutch Eating Behavior Questionnaire (DEBQ) for assessment of restrained, emotional, and external eating behavior. Int J Eat Disord. 1986;5(2):295–315. 10.1002/1098-108X(198602)5:2<295::AID-EAT2260050209>3.0.CO;2-T

[23] Bozan N, Baş M, Aşçı FH. Psychometric properties of Turkish version of Dutch Eating Behaviour Questionnaire (DEBQ). A preliminary results. Appetite. 2011;56(2):564–6. 10.1016/j.appet.2011.01.025. 21277923 10.1016/j.appet.2011.01.025

[24] Framson C, Kristal AR, Schenk JM, Littman AJ, Zeliadt S, Benitez D. Development and validation of the mindful eating questionnaire. J Am Diet Assoc. 2009;109(8):1439–44. 10.1016/j.jada.2009.05.006. 19631053 10.1016/j.jada.2009.05.006 PMC2734460

[25] Köse G, Tayfur M, Birincioğlu İ, Dönmez A. Yeme Farkındalığı Ölçeği’nin Türkçeye uyarlama çalışması. Bilissel Davranisci Psikoter Arast Derg. 2016;3(3):125–34. 10.5455/JCBPR.250644.

[26] Cohen J. Statistical power analysis for the behavioral sciences. 2nd ed. Hillsdale (NJ): Lawrence Erlbaum Associates; 1988.

[27] Sharma P, Rani MU. Effect of digital nutrition education intervention on the nutritional knowledge levels of information technology professionals. Ecol Food Nutr. 2016;55(5):442–55. 10.1080/03670244.2016.1198762. 27454492 10.1080/03670244.2016.1207068

[28] Ganasegeran K, Al-Dubai SA, Qureshi AM, et al. Social and psychological factors affecting eating habits among university students in a Malaysian medical school: a cross-sectional study. Nutr J. 2012;11:48. 10.1186/1475-2891-11-48. 22809556 10.1186/1475-2891-11-48 PMC3418187

[29] Mason AE, Jhaveri K, Cohn M, Brewer JA. Testing a mobile mindful eating intervention targeting craving-related eating: feasibility and proof of concept. J Behav Med. 2018;41(2):160–73. 10.1007/s10865-017-9884-5. 28918456 10.1007/s10865-017-9884-5 PMC5844778

[30] Tazeoğlu A, Ayten Ş, Tazeoğlu DT. Evaluation of the eating behavior of university students with the Dutch Eating Behavior Questionnaire (DEBQ): the case of Osmaniye Korkut Ata University. Turk J Clin Lab. 2020;11(5):429–35.

[31] Yue Y, Liu Y, Wang K, Zhang Y, Guo L. Impact of nutrition education on the eating habits, nutrition knowledge and health outcomes of university students: a systematic review and meta-analysis. Nutrients. 2022;14(3):569. 10.3390/nu14030569. 35276928 10.3390/nu14030569 PMC8840379

[32] Raut S, KC D, Singh DR, et al. Effect of nutrition education intervention on nutrition knowledge, attitude, and diet quality among school-going adolescents: a quasi-experimental study. BMC Nutr. 2024;10:35. 10.1186/s40795-024-00850-0. 38414069 10.1186/s40795-024-00850-0 PMC10900745

[33] Park S, Lee SY, Lee K, Kim M. Effects of nutrition education with mindfulness-based eating awareness training on eating behaviors and self-efficacy among university students: a randomized controlled trial. Nutrients. 2021;13(11):3924. 10.3390/nu13113924. 34836179 10.3390/nu13113924 PMC8624753

[34] Beshara M, Hutchinson AD, Wilson C. Does mindfulness matter? Everyday mindfulness, mindful eating and self-reported serving size of energy-dense foods among women. Appetite. 2019;132:216–22. 10.1016/j.appet.2018.10.005. 10.1016/j.appet.2013.03.012 23548262

[35] Aktaç S, Sabuncular G, Kargin D, Gunes FE. Evaluation of nutrition knowledge of pregnant women before and after nutrition education according to sociodemographic characteristics. Ecol Food Nutr. 2018;57(6):441–55. 10.1080/03670244.2018.1508735. 30421984 10.1080/03670244.2018.1544561

[36] Arabbadvi Z, Khoshnood Z, Foroughameri G, Mazallahi M. Education as an effective strategy to promote nutritional knowledge, attitudes, and behaviors in street children. BMC Public Health. 2023;23(1):989. 10.1186/s12889-023-15968-5. 37245021 10.1186/s12889-023-15400-9 PMC10224757

[37] Boek S, Bianco-Simeral S, Chan K, Goto K. Gender and race are significant determinants of students’ food choices on a university campus. J Nutr Educ Behav. 2018;50(7):687–94. 10.1016/j.jneb.2018.04.002. 22607739 10.1016/j.jneb.2011.12.007

[38] Tapper K, Shaw C, Ilsley J, Hill AJ, Bond FW, Moore L. Exploratory randomised controlled trial of a mindfulness-based weight loss intervention for women. Appetite. 2018;123:181–90. 10.1016/j.appet.2017.12.019. 10.1016/j.appet.2008.11.012 19101598

[39] Gkintoni E, Vassilopoulos SP, Nikolaou G. Mindfulness-based cognitive therapy in clinical practice: a systematic review of neurocognitive outcomes and applications for mental health and well-being. J Clin Med. 2025;14(5):1703. 10.3390/jcm14051703. 40095733 10.3390/jcm14051703 PMC11900371

[40] Plotnikoff RC, Costigan SA, Williams RL, Hutchesson MJ, Kennedy SG, Robards SL, et al. Effectiveness of interventions targeting physical activity, nutrition and healthy weight for university and college students: a systematic review and meta-analysis. Int J Behav Nutr Phys Act. 2019;16(1):43. 10.1186/s12966-019-0797-7. 25890337 10.1186/s12966-015-0203-7 PMC4393577

[41] İbiş R, Öztürk A. Üniversite öğrencilerinde beslenme okuryazarlığı durumu ve obezite ile ilişkisi: Yozgat örneği. Gumushane Univ J Health Sci. 2023;12(2):700–12.

[42] Loucks EB, Kronish IM, Saadeh FB, et al. Adapted mindfulness training for interoception and adherence to the DASH diet: a phase 2 randomized clinical trial. JAMA Netw Open. 2023;6(11):e2339243. 10.1001/jamanetworkopen.2023.39243. 37917063 10.1001/jamanetworkopen.2023.39243 PMC10623198

[43] Warren JM, Smith N, Ashwell M. A structured literature review on the role of mindfulness, mindful eating and intuitive eating in changing eating behaviours: effectiveness and associated potential mechanisms. Nutr Res Rev. 2017;30(2):272–83. 10.1017/S0954422417000154. 28718396 10.1017/S0954422417000154

[44] Chung LMY, Fong SSM. Role of behavioural feedback in nutrition education for enhancing nutrition knowledge and improving nutritional behaviour among adolescents. Asia Pac J Clin Nutr. 2018;27(2):466–72. 10.6133/apjcn.042017.03. 29384337 10.6133/apjcn.042017.03

[45] Mangwane QEM, Egal A, Oosthuizen D. Impact of a nutrition knowledge intervention on knowledge and food behaviour of women within a rural community. Nutrients. 2024;16(23):4107. 10.3390/nu16234107. 39683501 10.3390/nu16234107 PMC11644418

[46] Hill D, Conner M, Bristow M, O’Connor DB. Daily stress and eating behaviors in adolescents and young adults: investigating the role of cortisol reactivity and eating styles. Psychoneuroendocrinology. 2023;153:106105. 10.1016/j.psyneuen.2023.106105. 37028138 10.1016/j.psyneuen.2023.106105

[47] Dalton ED. Emotional eating in college students: associations with coping and healthy eating motivators and barriers. Int J Behav Med. 2024;31:563–72. 10.1007/s12529-023-10193-y. 37386338 10.1007/s12529-023-10193-y

[48] Ash S, Contento I, Olfert MD, Koch PA. Position of the Society for Nutrition Education and Behavior: nutrition educator competencies for promoting healthy individuals, communities, and food systems: rationale and application. J Nutr Educ Behav. 2023;55(1):P3–15. 10.1016/j.jneb.2022.07.010. 10.1016/j.jneb.2022.07.010 36372661

[49] Winpenny EM, van Sluijs EMF, White M, Klepp KI, Wold B, Lien N. Changes in diet through adolescence and early adulthood: longitudinal trajectories and association with key life transitions. Int J Behav Nutr Phys Act. 2018;15:86. 10.1186/s12966-018-0719-8. 30200990 10.1186/s12966-018-0719-8 PMC6131755

[50] Arrazat L, Nicklaus S, de Lauzon-Guillain B, Marty L. Behavioural determinants of healthy and environmentally friendly diets in French university students. Appetite. 2024;200:107532. 10.1016/j.appet.2024.107532. 38815688 10.1016/j.appet.2024.107532

