# Supplementary material for: Changes in mindful eating and eating behaviors among female university students taking nutrition courses
Source: J Eat Disord. 2026 May 4;14:141. doi: 10.1186/s40337-026-01624-8 (PMC13285342; doi:10.1186/s40337-026-01624-8)
Supplement: Supplementary file 1 — Supplementary Material 1. [file 40337_2026_1624_MOESM1_ESM.docx]

**Supplementary Table S1. Overview of the 14-week nutrition education course**

| **Week** | **Main topic** | **Key content** | **Behavior-focused component** |
| --- | --- | --- | --- |
| 1 | Nutrition basics | Basic concepts of nutrition, food groups, balanced diet, and the relationship between nutrition and health | Awareness of current eating habits |
| 2 | Carbohydrates, proteins, and fats | Functions and food sources of macronutrients, energy balance, and meal composition | Building balanced meals |
| 3 | Vitamins and minerals | Major micronutrients, deficiency/excess, food sources, and role in health maintenance | Recognizing nutrient quality in daily food choices |
| 4 | Nutrition across the life cycle | Nutritional needs during pregnancy, infancy, childhood, adolescence, adulthood, and older age | Understanding changing nutritional needs over time |
| 5 | Importance of nutrition in disease prevention and management | The role of nutrition in preventing chronic diseases and supporting treatment processes | Linking dietary choices with health outcomes |
| 6 | Eating behavior and determinants of food intake | Hunger, satiety, food choice, and environmental and psychological influences on eating | Increasing awareness of internal and external eating cues |
| 7 | Emotional eating, external eating, and restrained eating | Definitions, examples, and their relationship with daily eating patterns | Identifying maladaptive eating tendencies |
| 8 | Mindful eating | Awareness during eating, internal cues, eating pace, and attention to hunger and satiety | Improving eating awareness and self-regulation |
| 9 | Healthy meal planning and portion control | Meal timing, portion size, and practical organization of daily eating | Developing structured and balanced eating habits |
| 10 | Nutrition literacy and food label reading | Interpreting food labels, evaluating packaged foods, and making informed food choices | Supporting informed decision-making |
| 11 | Physical activity and nutrition | Relationship between physical activity and nutrition, energy needs, and lifestyle balance | Integrating nutrition and active living |
| 12 | Food science and everyday food choices | Food processing, practical food selection, and evaluation of common dietary habits | Translating nutrition knowledge into daily practice |
| 13 | Current issues in eating behavior | Discussion of selected scientific papers related to eating behavior and nutrition | Encouraging critical thinking and reflection |
| 14 | General review and integration | Review of course topics and linking knowledge with everyday eating awareness and behavior | Consolidating sustainable eating-related awareness |

**Note:** Each session concluded with the discussion of a scientific article relevant to the weekly topic, aimed at reinforcing content and promoting scientific literacy and critical thinking.

**Teaching approach:** The course combined theoretical instruction with interactive discussion, practical examples, and reflection on real-life eating situations.
