# Supplementary material for: Changes in mindful eating and eating behaviors among female university students taking nutrition courses
Source: J Eat Disord. 2026 May 4;14:141. doi: 10.1186/s40337-026-01624-8 (PMC13285342; doi:10.1186/s40337-026-01624-8)
Supplement: Supplementary file 2 — Supplementary Material 2. [file 40337_2026_1624_MOESM2_ESM.docx]

# Supplementary Material S2. General Information Form (Summary)

The general information form used in this study included items covering the following domains:

• Sociodemographic characteristics (age, sex, department of study)

• Anthropometric data (self-reported height and body weight)

• Health status (physician-diagnosed conditions and medication use)

• Lifestyle factors (smoking status, alcohol consumption, sleep duration)

• Dietary habits (meal frequency, meal skipping, water intake, dietary practices)

The questionnaire consisted of structured, self-reported items administered in the classroom setting. All data were collected for research purposes only and handled confidentially.

The full version of the questionnaire is available from the corresponding author upon reasonable request.
